# Synthetic Biology Applied to Carbon Conservative and Carbon Dioxide Recycling Pathways

**DOI:** 10.3389/fbioe.2019.00446

**Published:** 2020-01-10

**Authors:** Jean Marie François, Cléa Lachaux, Nicolas Morin

**Affiliations:** ^1^Toulouse Biotechnology Institute (TBI), Université de Toulouse, CNRS, INRA, INSA, Toulouse, France; ^2^Toulouse White Biotechnology Center (TWB), Ramonville-Saint-Agne, France

**Keywords:** microbial physiology, metabolic engineering, synthetic biology, carbon dioxide, bio-based products, chemicals

## Abstract

The global warming conjugated with our reliance to petrol derived processes and products have raised strong concern about the future of our planet, asking urgently to find sustainable substitute solutions to decrease this reliance and annihilate this climate change mainly due to excess of CO_2_ emission. In this regard, the exploitation of microorganisms as microbial cell factories able to convert non-edible but renewable carbon sources into biofuels and commodity chemicals appears as an attractive solution. However, there is still a long way to go to make this solution economically viable and to introduce the use of microorganisms as one of the motor of the forthcoming bio-based economy. In this review, we address a scientific issue that must be challenged in order to improve the value of microbial organisms as cell factories. This issue is related to the capability of microbial systems to optimize carbon conservation during their metabolic processes. This initiative, which can be addressed nowadays using the advances in Synthetic Biology, should lead to an increase in products yield per carbon assimilated which is a key performance indice in biotechnological processes, as well as to indirectly contribute to a reduction of CO_2_ emission.

## Introduction

**Figure 1:**
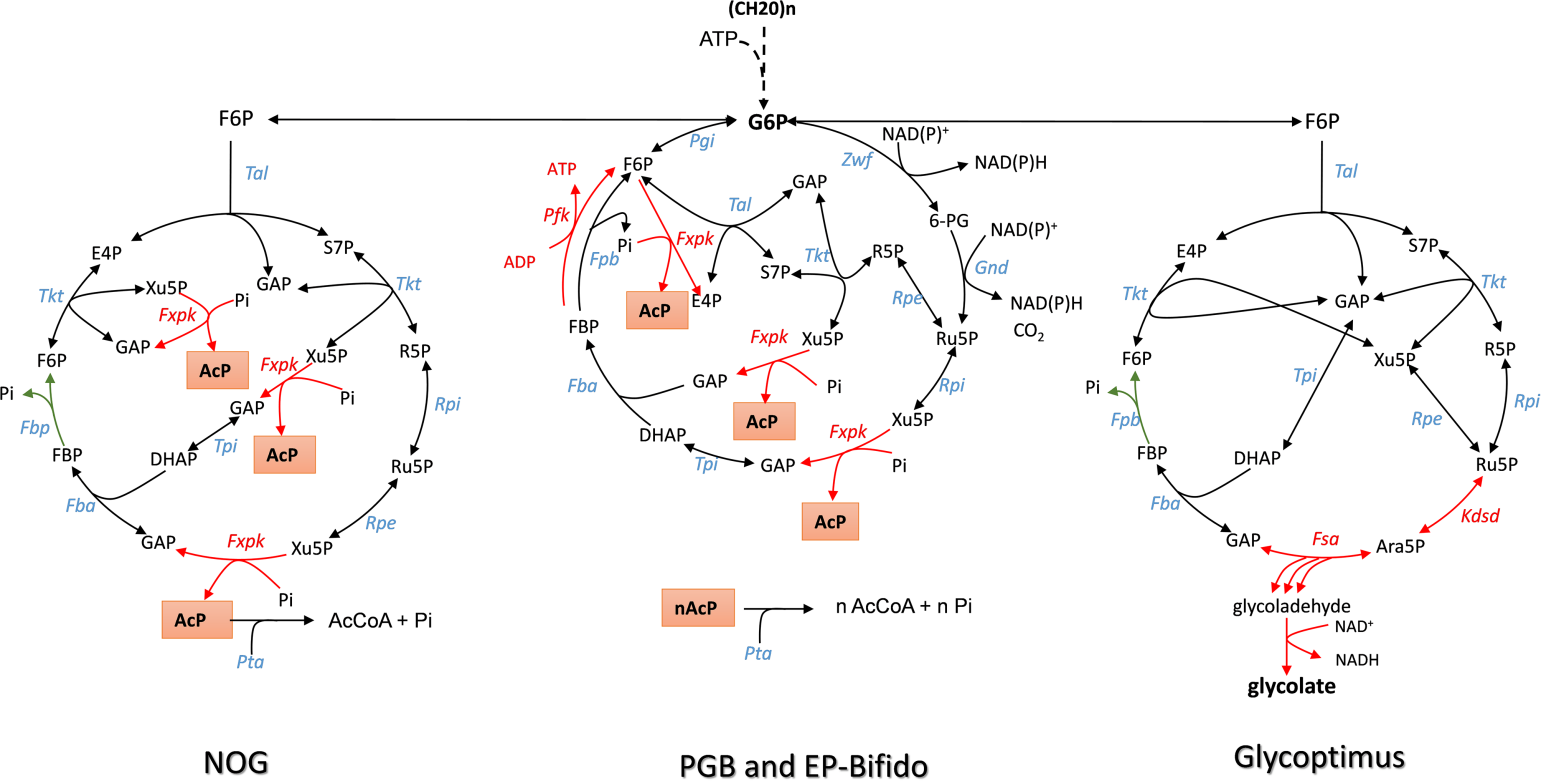
The scheme of non–oxidative glycolysis (NOG), Embden-Pentose-Bifido (EP-Bifido), and Glycoptimus synthetic pathways. Enzymes abbreviation are Fxpk, F6P/Xu5P-phosphoketolase; Tal, transaldolase; Tkt, transketolase; Fbp, fructose-1,6- bisphosphatase; Fba, fructose1,6 bisphosphate aldolase; tpi, triose phosphate isomerase; Rpe, ribulose 5-P epimerase; Rpi, ribose 5-P isomerase; Kdsd, arabinose-5-P isomerase; Fsa, fructose-6P aldolase; Alda, glycolaldehyde dehydrogenase. Metabolites: F6P, fructose-6P; PEP, phosphoenolpyruvate; Pyr, pyruvate; E4P, erythrose-4P; S7P, sedoheptulose-7P; R5P, ribose-5P; Ru5P, ribulose-5P; X5P, xylulose-5P; GAP, glyceraldehyde-3P; DHAP, dihydroxyacetone-P; FBP, fructose-1,6-bisphosphate; AcP, acetyl-Pi, AcCoA, acetyl-CoA.

The global warming is caused by the anthropogenic emission of greenhouse gases (GHGS), among which emission of carbon dioxide (CO_2_) is broadly accepted by the scientific community as the most contributing factor to this climate change. While Nature has been orchestrated with a natural carbon cycle, the industrial revolution that begun in the early 19th century resulted in a rise of CO_2_ emission that slowly but definitively exceeded the natural geochemical carbon cycle. According to the Keeling curve, which reports the daily carbon dioxide measurements at Mauna Loa observatory, the CO_2_ emission has dramatically increased over the last 50 years, mostly due to deforestation and burning of fossil fuels (https://scripps.ucsd.edu/programs/keelingcurve/). These issues are now becoming critical for the human being, which urged the scientific community to find solutions to decrease our reliance on fossil fuels that should eventually change our life style. Biofuels and more generally biorefinery have emerged as promising solution whose purpose is to exploit microorganisms as cell factories to convert non-edible but renewable carbon sources such as lignocellulosic sugars into bioethanol as alternative energy (Duwe et al., 2019; Rosales-Calderon and Arantes, 2019) or into commodity chemicals that can replace those obtained from petrochemistry (Clark et al., 2012; Chen and Dou, 2016; Singhvi and Gokhale, 2019). In spite of the apparent sustainability of this solution, the use of chemoorganotrophic microbes such as yeast or *E. coli* present a caveat, as CO_2_ is released during the process of carbon metabolism (Figure 1). A wonderful alternative would be to exploit autotrophic microorganisms such as acetogenic anaerobic bacteria or microalgae (Schiel-Bengelsdorf and Durre, 2012; Scaife et al., 2015) as they have the capability to capture atmospheric CO_2_. However, these biological systems are at the moment industrially inefficient, due to their slow growth, poor productivity and low energy conversion yield (Claassens, 2017). The purpose of this mini-review is to expose and discuss original strategies that have emerged recently and that have employed synthetic biology tools to rewire the carbon metabolism of heterotrophic microorganisms to achieve maximal carbon conservation during their metabolism. Basically, this endeavor can be reached by either redesigning carbon metabolism network to optimize carbon conservation or recapturing carbon loss using CO_2_ fixing or carboxylating systems. Autotrophic CO_2_ fixation by reductive pentose phosphate cycle (rPP also known as Calvin-Benson cycle) as well as other natural metabolic pathways that perform carbon fixation including reductive TCA cycle, 3-hydroxypropionate cycle, 3-hydroxypropionate/4-hydroxybutyrate cycle, and dicarboxylate/4-hydroxybutyrate cycle are not considered in this mini-review, since they have been extensively reviewed in previous excellent papers (Berg et al., 2010; Fuchs and Berg, 2014; Erb and Zarzycki, 2018).

## Rewiring Central Carbon Metabolism of Heterotrophic Organisms for Carbon Conservation

### Complete Carbon Conservation of Sugar Metabolism by the Non-oxidative Glycolysis Pathway

Glycolysis or the Embden-Meyerhof-Parnas pathway (EMP) is a fundamental metabolic pathway in most living systems that decomposes sugars into pyruvate and recovers energy of this breakdown into ATP and reducing equivalents NADH. To fuel the cell with some essential anabolic precursors, pyruvate has to be decarboxylated into acetyl-CoA. This decarboxylation step releases carbon dioxide in the environment, resulting in 33% loss of carbon yield. This wasted CO_2_ may have major impact on the overall economy of bio-based products derived from fermentable carbon sources. This 33% carbon loss due to the decarboxylation of pyruvate has been challenged recently by Liao's group (Bogorad et al., 2013) who constructed a synthetic pathway termed “the non-oxidative glycolysis (NOG)” that can overcome this carbon loss, leading to a conversion of one mole of glucose to 3 moles of acetyl-moieties (Figure 1 and Table 1 for the stoichiometric equation). The logic of NOG relies on the phosphorylating cleavage of sugar phosphates by a phosphoketolase (Fxpk), which, combined with a carbon rearrangement cycles, creates a cyclic pathway with F6P as the input molecule (Figure 1). The irreversibility of this pathway is ensured by the phosphoketolase reaction that cleaves Xu5P into glyceraldehyde-3-P (GAP) and AcP and by the fructose-1,6-bisphosphatase reaction (Fbp) which recycles back F6P from FBP. The carbon rearrangement also involves transaldolase (Tal) and transketolase (Tkt) and the net result of this FBP-dependent cycle is the irreversible formation of three acetyl-phosphate (AcP) molecules from one F6P molecule. As phosphoketolase also displays phosphorylating cleavage activity on F6P (Tittmann, 2014), cleaving the latter molecule into AcP and E4P, a sedoheptulose bisphosphate (SBP)-dependent network can be conceived yielding to the same solution. In this case, this cycle will not need transaldolase (*tal* gene) but will require a SBP-aldolase to condense DHAP and E4P into SBP, and a sedoheptulose phosphatase (Sbp) to recycle back S7P. Such cycle can potentially exist since Sbp is essential in the Calvin-Benson cycle, and has been identified in yeast as being implicated in ribogenesis (Clasquin et al., 2011) and in methylotrophic bacteria where it participates in the ribulose 5-monophosphate (RuMP) cycle (Stolzenberger et al., 2013).

**Table 1:** Stoichiometric equation of carbon conservative pathways described in Figures 1–**5**, and their calculated thermodynamic value^*^.

**Pathway name**	**Equation**	Δ_r_G^′^O^*^ (KJ/mole)
NOG	Glucose + 1 ATP + 3 CoaSH -> 3 acetyl-CoA + 1 ADP + 1 Pi	−193.6
PGB	Glucose + 4 NAD(P)^+^ + 2 CoASH -> 2 acetyl-CoA + 2CO_2_ + 4 NAD(P)H + 4 H^+^	−204
EP-Bifido	Glucose + 2 NAD(P)^+^ + 1 ATP + 2.5 CoASH + H_2_O -> 2.5 acetyl-CoA + 1 CO_2_ + 1 ADP + 1 Pi + 2 NAD(P)H + 2H^+^	−180.6
rGS	Glucose + 1 NAD^+^ + 1 ADP + 1 QH_2_ + 1 Pi + 3 CoASH -> 3 acetyl-CoA + 1 NADH + 1 H+ + 1 ATP + 1 Quinone	−229
MCG	Glucose + 1 ADP + 1 Pi + 3 CoaSH -> 3 acetyl-CoA + 1 ATP + H_2_O	−193.6
Glycoptimus	Glucose + 1 ATP + 3 NAD^+^ + H_2_O -> 3 glycolic acid + 1 ADP + 1 Pi + 3 NADH + 3 H^+^	−139.6
MOG	Glucose + 2 ${\text{HCO}}_{3}^{-}$+ 3 ATP + 3 H_2_O -> 2 glyoxylate + 2 acetate + 3ADP +3 Pi	−233
Mser	Methanol + CO_2_ + 2H_2_O + 4 ATP + CoASH + NADP(H) + H^+^ -> Acetyl-CoA + 4ADP + 4 Pi + NAD(P)^+^	−153
HOB	Tetrahydrofolate+ 2 NAD(P)H + ${\text{HCo}}_{3}^{-}+$ 4H_2_O + 3ATP -> 5′-10-methylene tetrahydrofolate + 3 ADP + 3 Pi + 2 NAD(P)^+^	−63.5

* *Gibbs energy value of the reaction was calculated using eQuilibrator (at http://equilibrator.weizmann.ac.il/)*.

In their Nature report, Liao and coworkers succeeded in demonstrating the *in vitro* and *in vivo* functioning of NOG (Bogorad et al., 2013). The *in vitro* system required the core of eight enzymes, which were either purchased when commercially available or produced by expression in *E. coli*. This was the case for phosphoketolase which is encoded by f*xpk* gene in *Bifidobacterium adolescensis* (Yin et al., 2005) and for some enzymes of the pentose phosphate pathway (PPP). In accordance with the pathway model, F6P initially added at 10 mM was readily and completely converted into 30 mM AcP. To validate the *in vivo* pathway, they chose to use xylose as the initial carbon substrate, as the phosphotransferase-dependent uptake and phosphorylation of glucose is not compatible with NOG and also because xylose does not cause repression of the *fbp* encoding fructose-1,6-bisphosphatase (Sedivy et al., 1984), which is needed together with the overexpression of *fxpk*. Other enzymes of the NOG pathway were natively expressed on a high copy plasmid, including *pta* and *ackA* which codes for phosphate acetyl transferase and acetate kinase, respectively, and which convert AcP into acetate. In addition, competitive fermentative pathways were disabled (i.e., deletion of *ldhA, pflB, adhE*, and *frdBC* to prevent production of lactate, formate, ethanol, and succinate) and the experiment was carried out under anaerobic condition to avoid further oxidation of acetate in the TCA cycle. Under this condition, 2.2 moles of acetate per mole of xylose were produced by the engineered *E. coli* equipped with the NOG pathway. In theory, the maximal yield that can be obtained by this conversion process should be 2.5. This yield termed the thermodynamic feasibility “Y^E^” can be calculated as the ratio between the degree of reduction of the substrate γ_s_ to that of the product γ_p_. Y^E^ is independent of the stoichiometry of the pathway and provides yield limits of the thermochemical process whatsoever the followed pathway or the catalyst used (Dugar and Stephanopoulos, 2011). In this case, Y^E^ of acetate from xylose (γ_s_ = 20) is 2.5 since γ_p_ of acetate = 6, whereas the stoichiometric yield “Y^st^” is 1,66. Therefore, the efficiency of the NOG pathway which can be calculated as the ratio of Y^E^ to Y^st^ is 50% better than the classical EMP pathway. The fact that the *in vivo* conversion of xylose into acetate by the engineered *E. coli* expressing the NOG pathway only reached 88% of Y^E^ was due to some by-products formation, notably succinate.

According to the stoichiometric equation (Table 1), each mole of acetyl-CoA produced from glucose by NOG can be converted into acetate, which is accompanied by the production of one ATP. Therefore, a net balance of 2 ATP and 3 acetate molecules can be obtained from glucose by NOG. However, this pathway is not compatible for growth as it can generate neither the 12 metabolic building blocks nor the reducing power needed for synthetizing all cellular constituents. These features may explain why NOG has not been retained as a viable pathway during evolution. Nevertheless, in a second seminal paper published in PNAS, Liao's group succeeded in evolving a *E. coli* strain that could grow while relying on NOG for carbon catabolism (Lin et al., 2018). To achieve this challenge, they blocked glycolysis at the level of glyceraldehyde-3-P dehydrogenase and phosphoglycerate kinase by deleting the corresponding genes as well as the methylglyoxal and the Entner-Doudoroff pathways to prevent bypass of glucose into pyruvate. The glyoxylate shunt and gluconeogenesis was then upregulated to enable conversion of acetyl-CoA to metabolic intermediates required for growth, whereas the reducing power and ATP were derived from TCA, which could be done only under aerobic condition. Since these genetic interventions could potentially generate futile cycles, notably at the level of F6P/FBP and pyruvate/ acetate, *pfkA* gene encoding the major phosphofructokinase and *poxB* encoding pyruvate oxidase were deleted. The final strain termed PHL13 contained 10 genes deletion and the 2 overexpressed genes *pck* and *fxpk* encoding PEP carboxykinase and phosphoketolase. This engineered strain was still unable to grow on glucose unless acetate was present. It was therefore subjected to evolution by serial transfer to selective media and meanwhile, a quest for the limiting enzymes in the NOG cycle was carried out by devising a clever whole-pathway assay in crude extract of the engineered strains. Practically, the assay was to measure the rate of AcP production from F6P in a crude extract augmented with a mix of all purified NOG enzymes and determine the effect of removing a particular enzyme from this mix on AcP production. This study identified phosphoketolase as the most limiting enzyme followed by transketolase and transaldolase. Therefore, these genes were expressed on a high copy plasmid that also contained genes encoding the glucose permease (*glf*) from *Zymomonas mobilis*, the glucokinase (*glk*), and a AMP-insensitive PEP-activated FBPase (*glpX*) from *E. coli* (Donahue et al., 2000). Strain PHL13 that carried out this constructed plasmid was then subjected to further adaptive laboratory evolution (Mattanovich et al., 2014; Jang et al., 2019) to get rid of its dependence to acetate and rely only on glucose for growth. After further improvement of transketolase activity by the whole-pathway assay, these authors could isolate a slow growth colony NOG21 on glucose minimal medium, which was further evolved to yield a faster growing colony NOG26. Use of ^13^C glucose labeled on C3 and C4 confirmed that the growth was accomplished by NOG since acetate was labeled on its C1 and C2, as expected. Genome sequencing of this evolved strain unexpectedly unraveled a reduced expression of *fxpk* and *pck*. This result could be explained by a fitness response of the strain to the growth condition in order to avoid “kinetic traps” that would cause imbalance of the metabolic flux, as for instance by draining out all oxaloacetate from the TCA cycle or Xu5P from the NOG cycle. Other mutations were identified in the metabolic network such as a deletion of *pykF* encoding the major pyruvate kinase or mutation in *pts* genes, which likely reduced the PEP dependent phosphorylation of glucose, leaving thus more PEP for gluconeogenesis. All these genomic modifications likely resulted in a fine-tuning of pathway regulation that would be difficult to predict *a priori*.

From a biotechnological perspective, the potential of NOG is very limited unless this pathway is coupled with another to make compounds other than acetate. An example would be to establish a NOG- based reductive fermentation in which additional reducing agents such as molecular hydrogen is provided together with sugars. In this condition, 3 moles ethanol can be produced per mole of glucose which would increase by 50% the maximal yield from natural sugar fermentation pathway. The challenge here will be to express hydrogenase to allow the input of additional reducing equivalents. Another practical use of NOG shall be to combine with the Calvin-Benson-Bassham (CBB) cycle (Calvin, 1962) for the biosynthesis of acetyl-coA derived products, such as 1-butanol and fatty acids by autotrophic organisms (Liu et al., 2011). This metabolic rewiring could represent a 50% increase of CO_2_ into acetyl-CoA over the native pathway, concomitantly reducing the requirement of RuBisCO activity to reach the same carbon yield (Tcherkez et al., 2006).

### The Pentose-Bifido-Glycolysis Cycling Pathway to Solve the Lack of Reducing Power in NOG

**Figure 2:**
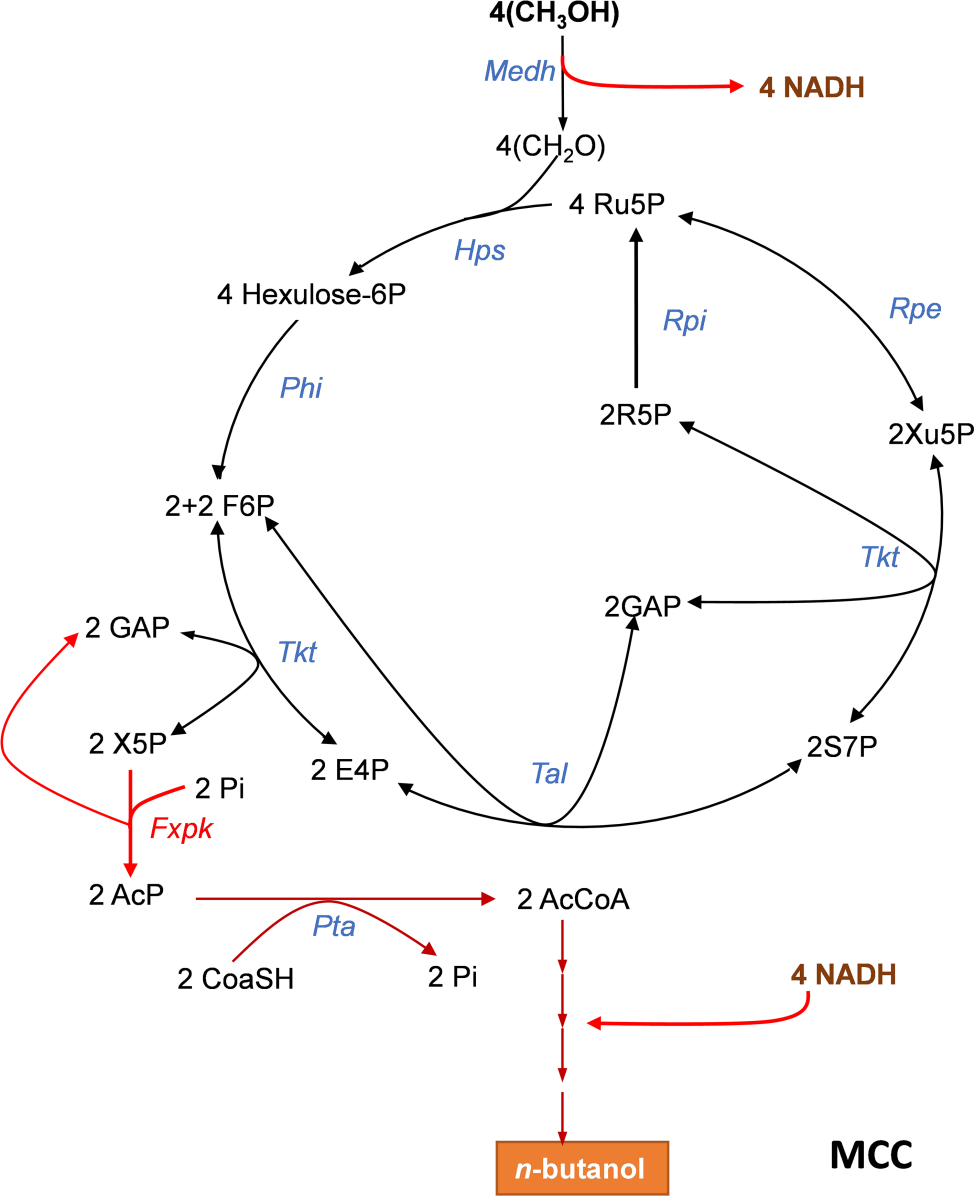
The methanol condensation cycle (MCC) for assimilation of methanol into higher alcohol with maximal carbon conservation. The MCC is a combination of the RuMP with part of the NOG allowing to avoid pyruvate decarboxylation and to bypass ATP dependency. It results in the net production of acetyl-CoA (AcCoA) from 2 methanol. Here, it is represented the metabolic pathway leading to the production of n-butanol which requires 4 methanol. Enzymes abbreviations are: Medh, NAD^+^-dependent methanol dehydrogenase; Hps, hexulose-6P synthase; Phi, phosphohexuloseisomerase; Tkt, transketolase, Fxkp, F6P/Xu5P phosphoketolase; Tal, transaldolase; Rpe, ribulose-5-P epimerase; Rpi, ribose-5-isomerase. Abbreviation for metabolites as in Figure 1.

A serious drawback of the NOG pathway is its inability to generate reducing power that is essential for the biosynthesis of added-value molecules from acetyl-CoA such as isoprene, fatty acids or polyhydroxybutyrate. A first approach to overcome this limitation was proposed by Opgenorth et al. (2016) who designed a synthetic *in vitro* pathway termed “Pentose-Bifido-Glycolysis” cycle (PBG) for the synthetic conversion of glucose into PHB via acetyl-CoA. This PBG cycle is divided in three metabolic parts. The first part is the phosphorylation of glucose into G6P which enters the cycle through the oxidative branch of the PPP and breaks down into Xu5P while producing NADPH. In a second phase, Xu5P is split into AcP and GAP by the phosphoketolase Fxpk. While AcP can be converted into acetyl-CoA by the CoA-phosphotransferase enzyme (Pta), GAP is recycled in a third phase into G6P. This occurs by the condensation of GAP and DHAP into FBP by the aldolase Fba, followed by a process involving a unique phosphofructokinase that catalyzes the ATP-dependent formation of F6P from FBP. At variance to the traditional phosphofructo-1-kinase reaction (Uyeda, 1979), this *E. coli* PFK encoded by *pfkB* was found to work reversibly and thus to regenerate ATP needed for glucose phosphorylation. This last phase enables to complete the cycle in ATP neutral manner. Accordingly, for each glucose entering the PBG cycle, two moles of acetyl-CoA, 2 moles of CO_2_ and 4 moles of NADPH is produced at zero cost of ATP (Table 1). However, there are two caveats with this synthetic pathway design. First the phosphoketolase Fxpk can also split F6P into E4P and AcP, which could abrogate the functioning of the cycle. Secondly, there is large excess of NADPH, as the synthesis of one PHB monomer only requires one NADPH, which therefore could lead to a feedback inhibition of the pathway. To solve the first issue, these authors (Opgenorth et al., 2016) complemented the pathway with PPP enzymes, namely transketolase (Tkt), transaldolase (Tal) and ribose-5-P isomerase (Rpi) in order to recycle E4P into the cycle (Figure 2B). The second problem was disentangled by introducing a “NAD(P)H purge valve” to regulate the build-up of NADPH. The outcome of this sophisticated purge valve is to uncouple carbon flux from NADPH production enabling a non-stoichiometric pathway production of NADPH. The construction of this purge valve requires the presence of NAD^+^-dependent glucose-6-P dehydrogenase (Zwf) and NAD^+^-dependent phosphogluconate dehydrogenase (Gnd) enzymes together with their native NADP^+^ dependent counterparts and a H_2_O-forming NADH oxidase (NoxE). While NADPH is reoxidized during PHB production, high level of NADPH would not penalize the cycle since it is taken over by the NAD^+^-dependent enzymes which continue to provide the precursor acetyl-CoA while NADH is readily reoxidized by NoxE. This original cell-free system production of bio-based chemicals from glucose turned out to be quite efficient as the authors reported a maximum productivity of the PBG cycle of 0.7 g PHB/L/h with a yield of 90%. There is great hope in developing such a process at an industrial scale since cell-free system can overcome common problems afflicting *in vivo* biological systems, such as building-up toxic intermediates, low productivities due to competing pathways and undesirable byproducts (Dudley et al., 2015). However, cell-free systems require to solve major technical and economic problems including production, purification, and stability of enzymes or their potential recycling.

Another approach to disentangle the failure of the NOG pathway to generate redox power was proposed by Wang et al. (2019) through the *in vivo* construction of an “EP-Bifido” pathway. This synthetic pathway is very similar to PGB as it combines EMP and PPP pathway with the expression of phosphoketolase enzymes acting on F6P and Xu5P, but it does not employ PFK reaction to regenerate the ATP that is needed to phosphorylate glucose (Figure 2B). As indicated in Figure 1, the major difference between NOG and the EP-Bifido is that the latter implicates the oxidative portion of the PPP, which leads to the production of NADPH. However, this metabolic process occurs at the expense of one CO_2_. The remaining reaction steps share those of the NOG with notably Fbp that provides the second driving force and recycles back F6P into the cycle. In theory, this pathway design should yield 2.5 acetyl CoA per mole glucose with two moles of NAD(P)H at the cost of 1 ATP (see Table 1). However, it may have the capacity to achieve an even higher yield of acetyl-CoA, as this will depend on favoring G6P into PPP with respect to EMP. In theory, 2.66 moles of acetyl-CoA could be produced from 1 glucose if 66% of G6P goes through PPP. To create this EP-Bifido pathway and expect to attain this optimal carbon yield, the following genetic modifications were carried out in *E. coli*. On the one hand, *zwf* and *gnd* were overexpressed to direct glucose into the PPP together with *fxpk* encoding F6P/Xu5P phosphoketolase from *Bifidus adolescentis*. On the other hand, the engineered strain was deleted for *pfkA* encoding the major phosphofructokinase, *edd/eda* encoding enzymes of the Entner-Doudoroff and *ackA* encoding acetate kinase to avoid, respectively, ATP wasteful by FBP/F6Pcycle, the production of pyruvate that could bypass the EP-Bifido pathway and the loss of AcP into acetate. Altogether, the engineered *E. coli* strain equipped with the EF-Bifido pathway grew 3 times slower than the wild type strain, did not produce any acetate and released 50 to 70% less CO_2_ than the wild type strain. The performance of the EF-Bifido pathway in acyl-CoA derived polydroxybutyrate (PHB), fatty acids or mevalonate was moreover demonstrated by showing that the yield of these products was improved by 145, 56, and 48% respectively in engineered strains as compared to the control strains (Wang et al., 2019), arguing for a biotechnological relevance of reengineering the carbon metabolism of *E. coli* with this pathway.

### The Glycoptimus Pathway to Convert Sugars Into Glycolic Acid Without Carbon Loss

While the refactoring of central carbon metabolic pathway was devoted to maximal acetyl-moieties from glucose, the glycoptimus pathway deals with a rewiring of the carbon metabolism to achieve maximal yield of glycolic acid (GA) from C5 and C6 sugars without carbon loss (Figure 1). According to thermodynamic calculation (Dugar and Stephanopoulos, 2011), the Y^E^ of glycolic acid (γ_p_ = 6) from glucose (γs = 24) or from pentose (γ_p_ = 20) should be 4 and 3.3, respectively, whereas the maximal yield Y^S^ based on the pathway stoichiometry is 2 from glucose and 1.66 from pentose (Dolan and Welch, 2018). Using the natural pathway, no <13 genetic modifications were executed to achieve a production of 52 g/L glycolate from glucose at a yield of 50 % of the Y^st^ (Soucaille, 2007). Further genetic modifications were proposed by Deng et al. (2018), by combining Dahms pathway with the glyoxylate shunt, enabling a yield close to 90% of the stoichiometric yield. The problem to reach the thermodynamic Y^E^ lies at two levels. A first one is to avoid the loss of CO_2_ at the pyruvate decarboxylation step, while the second is more challenging as it requires to capture one carbon mole as CO_2_. Since direct CO_2_ fixation by heterotrophic system is still very complicated as experienced by Milo and coworkers in *E. coli* (Antonovsky et al., 2016), we rewired the carbon central metabolism of *E. coli* in order to overcome the loss of CO_2_ during the conversion of sugars into glycolic acid. Theoretically, the proposed pathway should lead to a maximal yield of 3 GA per glucose (Table 1) or 2.5 GA per pentose, which is 50% higher than obtained by the natural pathway (Figure 1). This pathway design implicates the overexpression of *kdsD* and *fsaA* of *E. coli* which codes for an arabinose-5P (Ara5P) isomerase and a class I aldolase, respectively. Even though these two genes are endogenous to *E. coli*, this pathway is not natural in this bacteria because the Kdsd enzyme is naturally implicated in the synthesis of 2-keto-3-deoxy-octulosonate (KDO), a constituent of the outer member of cell wall lipopolysaccharide (Lim and Cohen, 1966), whereas Fsa has been initially reported as a F6P aldolase that catalyzes the aldol cleavage of F6P into dihydroxyacetone (DHA) and glyceraldehyde-3-P (GAP) (Schurmann and Sprenger, 2001). The bacterium *E. coli* also harbors an ortholog of *fsaA* termed *talC* or *fsaB* (Reizer et al., 1995) but the physiological function of these two genes is still completely unknown. In particular they are not expressed under standard—LB- culture condition (Schurmann and Sprenger, 2001). Nevertheless, Clapes' team demonstrated the remarkable originality of Fsa enzyme as a unique biocatalyst for asymmetric cross-aldol addition of glycoladehyde. They also reported that Fsa can cleave Ara5P into glycolaldehyde and GAP with a 10 fold higher affinity than F6P (Garrabou et al., 2009), which was confirmed in Lachaux et al. (2019). Therefore, the consecutive action of Kdsd and Fsa leads to the conversion of pentose phosphate intermediate Ru5P into glycolaldehyde and GAP (Figure 1). While glycolaldehyde can be oxidized into glycolic acid by an aldehyde dehydrogenase encoded by *aldA* (Caballero et al., 1983), GAP is recycled into Ru5P through the non-oxidative pentose phosphate pathway (Figure 1). This cycling scheme may result in theory in the production of 3 moles of GA per mole of glucose, only if the lower part of the glycolysis at the level of GAP is blocked. It also cost one ATP but generates 3 moles of NADH (Table 1) that needs to be reoxidized to allow the continuous functioning of the cycle. The reoxidation of NADH requires an active respiratory chain which is coupled to the synthesis of ATP. This ATP provision will be useful to regenerate ATP used in glucose phosphorylation, for uptake of sugars as well as for cellular maintenance. The *in vitro* and *in vivo* function of the glycoptimus pathway was validated although the yield of GA from glucose and xylose did not exceed 30% of the maximal yield expected. Possible explanation of this poor efficiency of the pathway could be that (i) the blockage of the lower part of glycolysis by deletion of *gapA* encoding GADPH was detrimental for cell viability; (ii) all competitive pathways using the same intermediaries have not yet been dismantled in the engineered strains; and (iii) the carbon flux in the *kdsD-fsaA* pathway is not optimized yet (Lachaux et al., 2019). Finally, the reoxidation of NADH is likely to generate more ATP than needed for the functioning of the glycoptimus cycle, which may result in feedback inhibition due to accumulation of intermediates and eventually depletion of cofactors. Solving these various issues together with the development of an appropriate fermentation practice should lead to an economically viable process of glycolic acid synthesis.

### The Methanol Condensation Cycle for Methanol Assimilation Without Carbon Loss

Abundant carbon sources other than sugars, such as methanol can be also exploited for the biosynthesis of added-value molecules. However, assimilation of these alternative resources also poses problem with carbon conversation. As an example, the ribulose-5- monophosphate pathway (RuMP) that is present in methylotrophic bacteria and in some yeasts such as *Pichia pastoris* (Yurimoto et al., 2002; Muller et al., 2015; Russmayer et al., 2015) is one of the three natural methanol assimilatory pathways known so far (Zhang et al., 2017) which can produce one acetyl-CoA from three formaldehydes at the expense of the decarboxylation of pyruvate. Liao's team (Bogorad et al., 2014) showed that the decarboxylation step could be avoided by combining the RuMP with phosphoketolase enzymes, leading to the production of acetyl-CoA from 2 formaldehydes through a methanol condensation cycle (MCC) as depicted in Figure 2. However, this MCC can only work if the thermodynamically unfavorable NAD^+^-dependent methanol dehydrogenase is coupled to the reoxidation of NADH into reduced products such as ethanol, n-butanol or even higher alcohols (Bogorad et al., 2014). Remarkably, the MCC reaction is completely redox balanced and independent of ATP, and thus does not involve the phosphofructokinase enzyme of the RuMP, as well as the triose phosphate isomerase (Tpi), fructose 1,6-bisphosphate aldolase (Fba) and fructose-1,6-bisphosphatase (Fbp) of the NOG pathway. This property has been exploited to demonstrate the *in vitro* functioning of MCC to the production of ethanol or n-butanol. Therefore, a cell-free system could be potentially exploitable for larger scale production of higher alcohols with maximal yield and at high productivity if conditions for enzymes and intermediates stability are assured. In addition, this *in vitro* study pointed out the role of phosphoketolase in the robustness of the MCC, showing that its optimal running requires well-balanced levels of this enzyme with respect to the others, as a low or excessive phosphoketolase activity could trigger kinetic traps that significantly diminish rate of end-product production, due to either accumulation or depletion of intermediates metabolites in the cycle.

## Carbon Conservation Synthetic Pathways Building on Carboxylating Reactions

### Reversal of the Glyoxylate Shunt to Build C2 Compounds With Maximal Carbon Conservation

**Figure 3:**
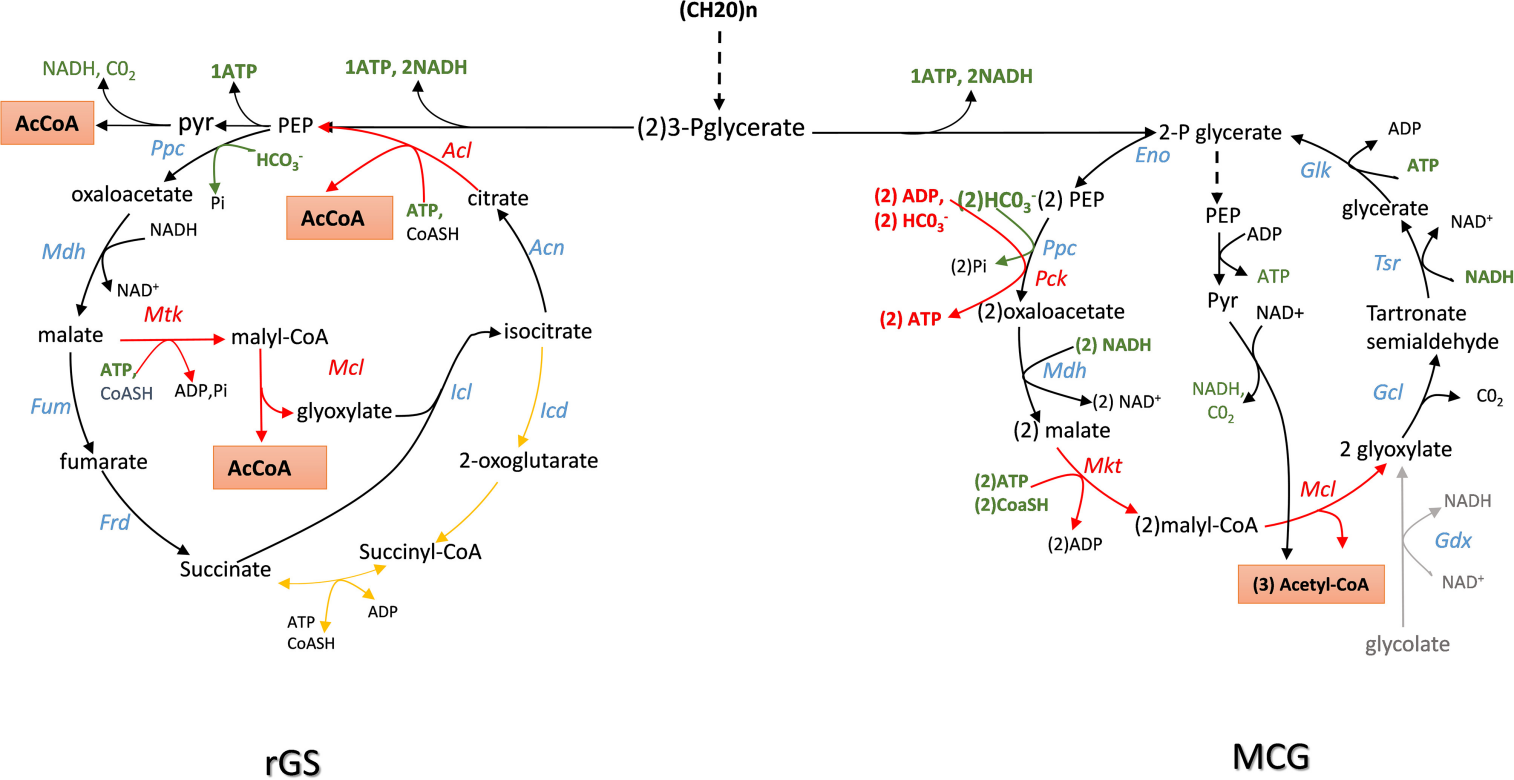
Scheme of the reversal of glyxoylate shunt (rGS) and maly-CoA Glycerate (MGC) cycling pathway for the production of acetyl-CoA from sugars with maximal carbon conservation. Enzymes abbreviation are: Ppc, PEP carboxylase; Mdh, malate dehydrogenase; Fum, fumarase; Frd, fumarate reductase; AceA, isocitrate lyase; Aco, aconitase; Acl, acyl-CoA lyase: Mtk, malate thiokinase; Mcl, malyl-CoA lyase; Gdh, glycolate dehydrogenase; Gcl, glyoxylate carboxyligase; Tsr, tartronate semialdehyde reductase; Glk, glycerate kinase; Eno, enolase.

The TCA or citric acid cycle elucidated by Krebs and Johnsson in 1937 (see Krebs and Johnson, 1980 for an historical perspective or Akram, 2014 for a recent review of TCA role in intermediary metabolism) is considered as the central metabolic hub of the cell whose amphibolic nature provides intermediates for numerous metabolic functions. This cycle generates energy and reducing power needed for anabolic activities of the cell at the expense of complete oxidation of pyruvate into carbon dioxide. The glyoxylate shunt, discovered by Krebs and Kornberg in 1957 (see Dolan and Welch, 2018) for an excellent review on this pathway) avoids the decarboxylation steps of the TCA cycle and allows acetyl-CoA to be converted into a C4 carbon without carbon loss. This shunt is therefore key for organisms that grow on C2 to C4 carbon such as acetate, glycerol and malate, or on ketogenic amino acids (i.e., aspartate, glutamate). The glyoxylate shunt takes carbon away from the TCA cycle at the level of isocitrate, just before the commencement of the first decarboxylation step. This requires two specific enzymes that are the hallmark of this cycle: isocitrate lyase that cleaves isocitrate into succinate (C4) and glyoxylate (C2), and malate synthase, which condenses glyoxylate with acetyl-CoA to malate. Due to the large negative Gibbs value of the malate synthase reaction (ΔrG'^0^ = −44 kJ/mol), the glyoxylate cycle can only run in the acetyl-CoA condensation. However, running the cycle clockwise directly could be beneficial to supply acetyl-CoA from sugars by bypassing the decarboxylation step of pyruvate. As illustrated in Figure 3, a reversal of the glyoxylate shunt (rGS) in which the thermodynamically favorable PEP carboxylating reaction is implemented could generate 2 acetyl-CoA from TCA intermediates. This challenge was raised by the Liao' group according to the following strategy (Mainguet et al., 2013). At first, they demonstrated that isocitrate lyase (ICL) is reversible *in vivo* by showing that a *E. coli* strain made auxotroph for glutamate (glu^−^ strain) by deletion of *gltA* and *prpC* encoding citrate synthase was able to grow on glucose mineral medium supplemented with glyoxylate and succinate. In contrast, this glu^−^ strain was unable to recover growth when glyoxylate was replaced by malate, even if *dctA* gene encoding a non-glucose repressible malate importer from *B. subtilis* had been overexpressed. These results confirmed that malate synthase reaction was not reversible *in vivo*. To get around this obstacle, they wished to express an ATP-dependent malate thiokinase (Mtk) encoded by *mtkA* and *mtkB* from *Methylobacterium extorquens*. In this methanotrophic bacterium, this enzyme contributes to C1 assimilation through the serine cycle (Fei et al., 2014). This reaction step is then followed by the cleavage of malyl-CoA into glyoxylate and acetyl-CoA by a malyl-coA lyase (Mcl) naturally present in microorganisms that use the 3-hydroxypropionate cycle for autotrophic carbon dioxide fixation (Berg et al., 2007). Unfortunately, the expression of *mtkAB* and *mcl* from *Methylobacterium extorquens* expressed in a glu^−^ strain did not rescue growth on malate and succinate likely because Mtk was not functional. They overcame this problem by screening various putative malate thiokinase and discovered that *sucCD-2* from *Methylococcus capsulatus* annotated as encoding a succinyl-CoA synthetase (Ward et al., 2004) had very good Mtk activity. Expression of *sucCD-2* gene with *mcl* restored the capacity of growth of a glu^−^ strain on glucose supplemented with malate and succinate. The second important action was to recycle OAA from isocitrate that is accompanied by the release of a second molecule of acetyl-coA. This involves to reverse the aconitase and the citrate synthase reaction. While the first one is readily reversible, the conversion of citrate to OAA by citrate synthase is strongly unfavorable (ΔrG'°~ + 34 KJ/mol). It can nevertheless be obtained by expressing an ATP-dependent citrate lyase encoded by *acl* which is present in several eukaryotic cells and in some archeabacteria (Fatland et al., 2000; Verschueren et al., 2019). The *in vivo* functionality of the Acl reaction was tested in *E. coli* strain made auxotroph to aspartate (asp^−^ strain) by deleting genes encoding all enzymes that produce its precursor OAA (ie *ppc, mdh, mqo*), as well as citrate synthase and citrate lyase (*gltA* and *citE*). As expected, the asp^−^ strain recovered growth on a glucose minimal medium when supplemented with citrate. Although the thermodynamically unfavorable reactions were solved to potentially create a reverse glyoxylate cycle, it was also necessary to remove isocitrate dehydrogenase (*icd*) to avoid siphoning isocitrate obtained by condensation of glyoxylate and succinate into α-ketoglutarate. Then, the assembly of the complete pathway in asp^−^ strain which was otherwise deleted for *mdh, mqo, ppc, icd*, and *aceB* consisted in the overexpression of Bs*dctA*, Mc*succD-2*, Rs*mcl*, and Ct*acl* genes. This strain was shown to grow on glucose supplemented with malate and succinate, demonstrating the *in vivo* function of this rGS. Until now, the complete demonstration of the “*in vivo*” reversal of the glyoxylate cycle has not been fully demonstrated. It will require to connect OAA to malate which can be easily carried out by the reversible NAD^+^-dependent malate dehydrogenase encoded by *mdh*. However, cycling malate shall require also expression of a fumarase (Fum) and a fumarate reductase (Frd), both enzymes being preferentially active under anaerobic conditions (Lin and Iuchi, 1991; Tseng, 1997).

In theory, the integration of rGS with the central carbon metabolic pathway via the PEP carboxylase encoded by *ppc* could lead to the conversion of one mole glucose into 3 moles of acetyl-CoA, thus achieving a 50% yield increase over the native pathway. Contrary to the NOG pathway that converts glucose to 3 AcCOA at the expense of one ATP, the conversion of glucose into AcCOA by the rGS generates one extra ATP. In addition, while NOG is redox neutral, the rGS pathway results in the net production of one NADH and one reduced quinone per glucose (Table 1). This indicates that rGS is more energetically efficient than NOG which could be an advantage with respect to the production of acetyl-CoA derived products such as isoprenoids, fatty acids, long chain alcohols as they required reduced power and ATP (Tabata and Hashimoto, 2004; Kondo et al., 2012; Robles-Rodriguez et al., 2018).

### The Malyl-CoA—Glycerate Cycling (MCG)

Like the rGS pathway, the synthetic Malyl-CoA-Glycerate (MCG) cycling pathway allows the conversion of glucose into acetyl-CoA. As depicted in Figure 3, this synthetic pathway relies on 6 natural reaction steps that are catalyzed by endogenous enzymes in *E. coli*, completed by the heterologous expression of *mtkAB* and *mcL* from methylotrophic bacteria. The importance of these two reaction steps in the *in vivo* functionality of the MCG was demonstrated by showing that the growth on glucose of an acetyl-COA auxotroph *E. coli* strain (Δ*aceE* Δ*poxB* Δ*plfB*) can be rescued upon the expression of *mtkAB* and *mcl* genes. Like NOG and rGS, the theoretical molar yield of acetyl-COA per glucose consumed is 3 (Table 1). Another interesting feature of the MCG is to be able to assimilate C2 compound such as glycolate or glyoxylate (Figure 3). Hence, in photosynthetic organisms, expression of MCG could improve carbon fixation in combination with the CBB cycle by re-assimilating glycolate produced by photorespiration. Also, the conversion of glucose into acetyl-CoA by the MCG is redox neutral and costs one ATP for glucose phosphorylation, unless the glycolytic PEP carboxykinase as it exists in the rumen bacterium *Actinobacillus succinogenes* (Kim et al., 2004; Leduc et al., 2005) is used instead of PPC, which would result in the production of one extra ATP.

## Exploiting Carboxylating Enzymes for CO_2_ Fixation in Heterotrophic Organisms

### Carboxylating Enzymes as a Key Reaction in Heterotrophic Organisms for CO_2_ Fixation

**Figure 4:**
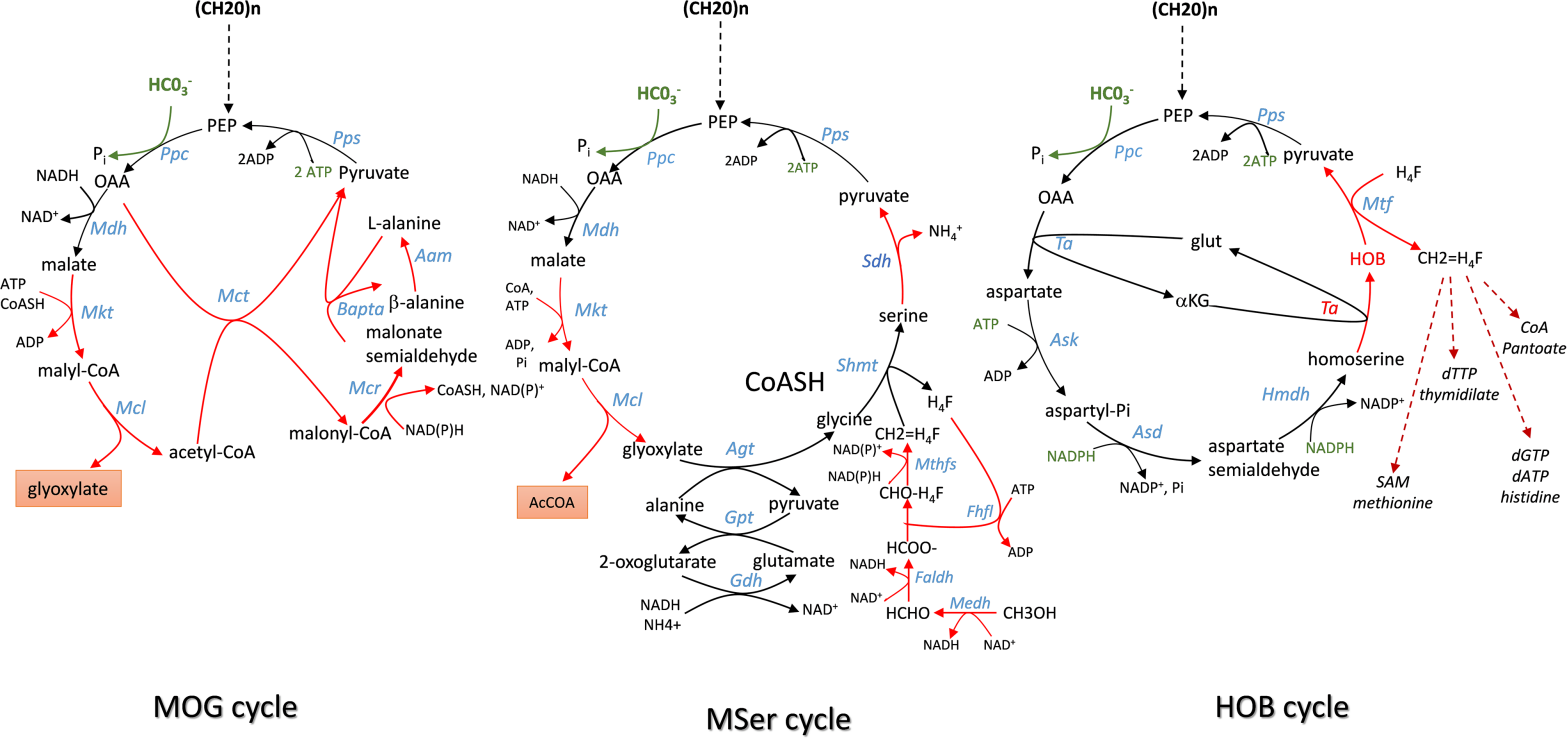
Scheme of Malonyl-coA-Oxaloacetate- Glyoxylate (MOG), Modified Serine (MSer) and 4-hydroxy-2-oxo-butyrate (HOB) cycling pathway employing the efficient PEP carboxylase to capture Co_2_ into either product or biomass. Enzymes abbreviation are: Ppc, PEP carboxylase; Mdh, malate dehydrogenase; Mtk, malate thiokinase; Mcl, malyl -CoA lyase; Mct, malonyl-CoA acetyl transferase; Mcr, malonyl CoA reductase; Bapta, β-alanine: pyruvate transaminase; Aam, alanine 2,3 aminomutase; Pps, PEP synthase; Ta, transaminase; Ask, aspartate kinase; Asd, aspartyl-Pi semialdehyde dehydrogenase; HmdH, homoserine dehydrogenase; Hmtf, hydroxymethyl transferase.

In an elegant paper published in 2010, Bar-Even et al. (2010) developed a constrained-based modeling approach that explored all possibilities that could be devised from a repertoire of ~5,000 known metabolic enzymes reported in the KEGG database (https://www.genome.jp/kegg/pathway.html) to generate carbon fixation pathways that are alternative to the 5 known to date. Criteria used in their modeling were (i) the enzyme's specific activity is equal or superior to that of RuBisCO which is used as the benchmark activity; (ii) a minimal energy cost of the pathway, which corresponds to the cost in NAD(P)H, ferredoxins, FADH_2_, and ATP in the production of one mole of products from CO_2_; (iii) thermodynamic feasibility of the pathway under a plausible physiological range of metabolites concentrations; (iv) topology of the pathway, which incorporates the number of enzymatic reactions the carbon fixation pathway can make as an independent unit and (v) the compatibility or integration of this synthetic pathway with the endogenous metabolic network. According to these criteria, they identified several cycling pathways containing four to six enzymatic steps leading to the production of the C2 compound glyoxylate. Although most of them were unrealistic because thermodynamically unfeasible or using oxygen-sensitive ferredoxin-oxidoreductase enzyme, these authors found that kinetically efficient pathways absolutely require a carboxylating enzyme with high activity and affinity toward CO_2_ or ${\text{HCO}}_{3}^{-}$ whose reaction must be physiologically irreversible. PEP carboxylase (*ppc* gene) and pyruvate carboxylase (*pyc* gene) came at first in their listing, followed by acetyl-CoA and propionyl-CoA carboxylase. Accordingly, they designed a pathway family termed Malonyl-CoA-Oxaloacetate-Glyoxylate (MOG) cycle employing either PEP carboxylase or pyruvate carboxylase as the sole carboxylating enzyme and that has a higher carboxylating activity than the reductive pentose pathway (rPP). This MOG pathway resembles the natural C4 cycle in which the recuperation of CO_2_ arising from malate decarboxylation to pyruvate by RuBisCO is replaced by a PEP or pyruvate carboxylating enzyme. The net product of MOG is the C2-carbon glyoxylate, which can be converted into GAP by the bacterial-like glycerate pathway (Eisenhut et al., 2006; Igamberdiev and Kleczkowski, 2018) (Figure 4). Interestingly, this pathway turns out to be thermodynamically feasible and more efficient than the rPP, although it has not been found in Nature yet. Moreover, combined with the plant natural C4-cycle, it could allow to overcome the futile CO_2_ cycling that is taking place in the bundle sheath cell and which is due to malate decarboxylation coupled to reassimilation of CO_2_ by RuBisCO. In addition, this coupling could result in an extra CO_2_ fixation with the release of glyoxylate. Therefore, it can be anticipated that the expression of MOG in autotrophic cells may provide great advantages from a biotechnological point of view, with faster growth and increased crop yields because of additional carbon dioxide input and absence of competing oxygenation reaction that reduces carbon fixation by plants. Whether the implementation of this MOG pathway in a heterotrophic cell like *E. coli* is biotechnologically relevant is less obvious. Indeed, this implementation will require the expression of heterologous genes encoding six out of the nine reactions of this cyclic pathway (Figure 4), namely the malate thiokinase (*mtkAB*), the malyl-CoA lyase (*mcl*), the methylmalonyl-COA carboxytransferase (*mct*), the malonyl-CoA reductase (*mcr*), the β-alanine-pyruvate transaminase (*bapta*), and the alanine 2,3 aminomutase *(aam*). More importantly, the stoichiometry evaluation of this route from glucose indicated that it can produce 2 moles of glyoxylate and 2 moles of acetate, with the requirement of two mole of CO_2_. However, while globally thermodynamically favorable and redox balanced, this reaction can occur at the expense of 3 ATP, which can be obtained by consumption of acetate, and hence resulting in the reemission of 2 moles of CO_2_. Therefore, this route has likely no meaning to be expressed in a chemoorganotrophic system.

### The Modified Serine Cycle Pathway in *E. coli*

**Figure 5:**
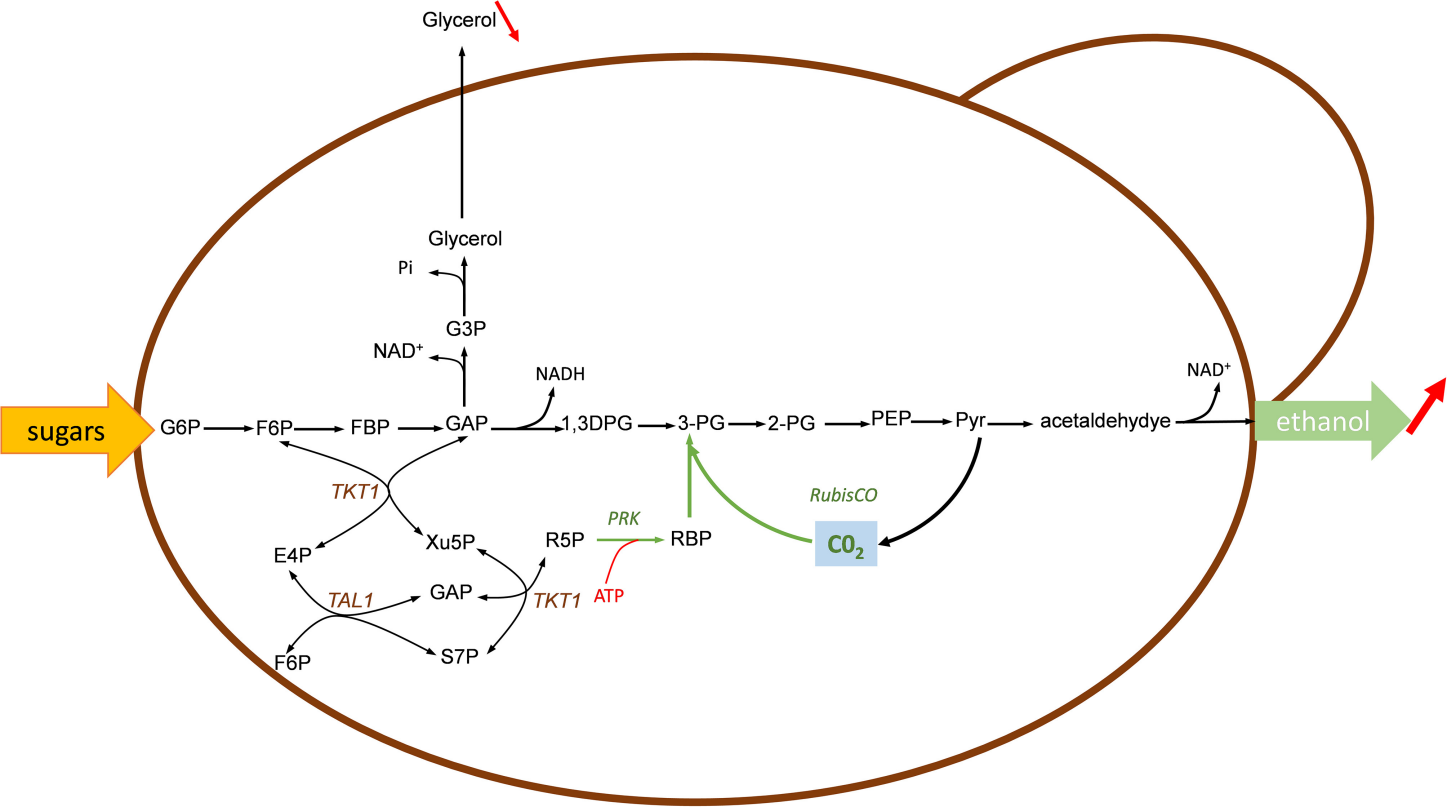
Engineering sugars fermentation with Calvin-cycle enzymes for fixing *in-situ* CO_2_ to improve bioethanol yield in yeast. In green are shown the Calvin-cycle enzymes corresponding to RuBisCO and phosphoribulose-5-P kinase (PRK). The expression of this Calvin-cycle enzymes enables the use of CO_2_ as alternative electron acceptor for reoxidation of NADH, thereafter reducing glycerol production under anaerobic condition. Enzymes: RuBisCO, ribulose 1,5 bisphosphate carboxylase; PRK, phosphoribulose 5-P kinase; Tkt, transketolase; Tal, transaldolase. Metabolites abbreviation, as in Figure 1 except: 1,3-DPG, 1,3 diphosphoglycerate; 3-PG, 3-phosphoglycerate; 2-PG, 2-phosphoglycerate; G3P, glycerol-3-P.

In methylotrophic bacteria, the serine cycle is a natural pathway to assimilate C1 carbon such as methanol or methane into acetyl-CoA intermediate without loss of carbon (Smejkalova et al., 2010). A modified serine cycle pathway has been designed in *E. coli* to allow co-assimilation of C1-carbon such as methanol or formate with bicarbonate to generate acetyl-COA as a precursor for several bio-based products (Yu and Liao, 2018). This synthetic pathway encompasses nine enzymatic reactions for the cycle in which CO_2_ is captured by the reaction catalyzed by PEP carboxylase, and four additional reaction enabling assimilation into 5–10 methylene tetrahydrofolate (CH_2_ = H_4_F). The methyl unit is afterwards transferred on serine cycle by a reaction involving glycine and catalyzed by serine hydroxymethyl transferase (Shmt). From these 13 enzymatic steps, seven (highlighted in red in Figure 5) had to be implemented using heterologous genes from various organisms, which included *S. cerevisiae* for the glyoxylate:alanine transaminase (Agt) encoded by *AGX1*, serine dehydratase (Sdh) from *Cupriavidus necator* encoded by *SdaA*, malate thiokinase (Mkt), and malyl-CoA lyase(Mcl) from *M.extorquens*. For methanol oxidation and its conversion to 5–10 methylene tetrahydrofolate, a variant of the *Cupriavidus necator* methanol dehydrogenase (Medh) with higher specific activity was used together with formate-H4F ligase (Fhlfl) and 5,10-methylene tetrahydrofolase synthase (Mthfs) from *Moorella thermoacetica*. The NAD^+^-linked formadehyde dehydrogenase (Faldh) from *Pseudomonas putida* was employed to oxidize formaldehyde to formate. All these genes were carried on 3 different plasmids and transformed into *E. coli* strain HY106, which was deleted for genes to avoid byproducts formation and shortage of intermediates in the cycle. Accordingly, *aceB* and *glcB* both encoding malate synthase were deleted to avoid competitive reverse reaction catalyzed by Mkt and Mcl. The *gcl* gene encoding glyoxylate carboxyligase was deleted to prevent shortage of glyoxylate into tartronate semialdehyde, as well as *gcvP* to avoid loss of glycine by decarboxylation. Genes encoding lactate dehydrogenase (*ldhA*) and fumarate reductase (*frABCD*) were also deleted in this strain to avoid shortage of pyruvate into D-lactate and malate into succinate. As indicated in Table 1, co-assimilation of methanol with CO_2_ needs 3 moles ATP and 1 mole reduced equivalent per mole of acetyl-CoA produced. Hence, it cannot proceed without addition of another carbon source to provide this energy and cofactors. Thus, the *in vivo* function of the complete cycle was demonstrated using C^13^-methanol together with xylose to supply energy and PEP. In addition the pyruvate to malate flux was increased upon overexpression of *pyc* and *mdh* encoding pyruvate carboxylase and malate dehydrogenase in the engineered *E. coli* equipped with the Mser pathway. The engineered strain could produce acetate from which 33% came from methanol. When the same experiment was carried out with both C^13^ methanol and C^13^ bicarbonate, the amount of acetate produced was the same but the labeling of both C1 and C2- carbon of acetyl-moieties was enriched by 2-fold, indicating that bicarbonate has been also incorporated into acetyl moieties. To conclude, the construction of this synthetic serine cycle in *E. coli* enables the assimilation of C1 compounds such as methanol to increase the production of C2-compounds as precursor of bio-based products such as PHB, isoprenoid, fatty acids, etc. In addition, this cycle, combined with the glyoxylate shunt, is capable to support growth from pyruvate that is formed from two C1-carbon (formate or methanol) and one bicarbonate, unless the mole excess of NAD(P)H is reoxidized by respiratory chain coupled with ATP.

### The HOB Pathway as an Alternative for C1-Carbon Metabolism Through CO_2_ Fixation by PEP Carboxylase

Another illustration about the potential function of carboxylating enzyme to incorporate CO_2_ into biomass comes from the work of Bouzon et al. (2017). These authors built a synthetic pathway that is alternative to the ubiquitous one carbon pathway transferring C1-moities (i.e., formyl, hydroxymethyl, or methyl group) from tetrahydrofolate for the synthesis of purine nucleotides, thymidylate, methionine, or coenzyme A (Figure 4). These C1-moities are obtained from the pivotal intermediate 5,10-methylene tetrathydrofolate (CH_2_=H_4_F) produced by the condensation of tetrahydrofolate with formaldehyde that originates from serine or glycine. To replace these amino acids as C1-donor, these authors devised a cyclic pathway in which the C1 compound (in this case formaldehyde) is obtained from the aldolytic cleavage of the non-natural intermediate 2-keto-4-hydroxybutyrate (HOB). As shown in Figure 4, the complete cycle encompasses 8 enzymatic steps in which the C1- source is ${\text{HCO}}_{3}^{-}$ that is captured by PEP carboxylase to yield OAA. Through a set of 4 natural enzymatic step, OAA is converted into homoserine which gives rise to HOB through a transaminase reaction. The best candidate for this non-natural transaminase reaction was obtained by employing an automated evolutionary technology originally developed by Marlière et al. for evolving *E. coli* possessing DNA in which thymine was replaced by the synthetic building block 5-chlorouracil (Marliere et al., 2011). The evolved transaminase was found to correspond to a variant of the alanine: pyruvate transaminase encoded by *alaC* having amino change at position 142 (Ala to Pro) and 245 (Tyr to Glu). The HOB is then cleaved into pyruvate and formaldehyde which is readily transferred into H4F by the promiscuous hydroxymethytransferase (Mtf) encoded by *panB*. The cycle is then closed by converting back pyruvate into PEP, which implicates PEP synthase. Overall, the functioning of this cycle costs two moles of NADPH and three moles of ATP per mole of CO_2_ incorporated (Table 1). To implement this synthetic pathway that completely rewires the C1-canonical metabolism by imposing HOB as the essential metabolic intermediate, the authors banked on an evolutionary trajectory of a *E. coli* strain lacking all enzymes necessary for the natural C1- transfer moieties using an original automated evolutionary technology that is now commercialized as “Heurisko” by the company Altar (http://www.altar.bio/). The authors estimated that the CO_2_ assimilated in the biomass of *E. coli* expressing this HOB pathway is about 2 times more than in wild type strain (Bouzon et al., 2017). However, being quite energy consuming, the relevance of this pathway in the production of biotechnological molecules methionine is questionable.

## Expression of the Calvin-Benson Cycle Enzymes for *in-situ* CO_2_ Reintegration Into the Central Metabolic Network

Under anaerobic condition, sugar fermentation by the yeast *Saccharomyces cerevisiae* into ethanol generated excess of NADH, which must be reoxidized to warrant redox-cofactor balancing (Van Dijken and Scheffers, 1986). This reoxidation diverts between 4 and 10% of the sugar into glycerol, which has a significant impact on the economy of large scale yeast-based bioethanol production. To reduce or even eliminate this by-product and in the same time, to increase the ethanol yield, the group of Jack Pronk in TU Delft proposed to use CO_2_ as electron acceptor for the reoxidation of this excess of NADH. A theoretical analysis indicates that the replacement of glycerol by CO_2_ can increase the ethanol yield from sugar by 14% if the CO_2_ is incorporated through phosphoribulokinase (PRK) and ribulose-1,5-bisphosphate carboxylase/oxygenase (RuBisCO) onto ribulose 5-P (Ru5P) (Figure 5). In their design, Ru5P must be produced from the non-oxidative pentose phosphate route in order to reoxidize all biosynthetic NADH and not NADPH through a transhydrogenase-type conversion NADPH-> NADH (Guadalupe-Medina et al., 2013). This clever idea was demonstrated in two elegant papers published in Biotechnology for Biofuels (Guadalupe-Medina et al., 2013; Papapetridis et al., 2018). In the first paper, the authors expressed the codon-optimized prokaryotic form II RuBisCO-encoding *cbbM* gene from *T. denitrificans* in a centromeric plasmid under the strong *TDH3* promoter and showed that RuBisCO was functional only upon co-expression of *E. coli groEL/groES* encoding chaperones whereas surprisingly *T denitrificans* chaperones encoded by *cbb02/cbbQ2* were ineffective. The *PRK* from *Spinachia oleracea* was integrated together with the *E. coli groEL/groES* gene in the yeast genome at the *CAN1* locus under the galactose-inducible *GAL1* promoter. Remarkably, expression of this minimal Calvin cycle in this engineered yeast cultivated under anaerobic chemostat condition showed a 68% reduction of glycerol production accompanied by a 10% increase of ethanol yield. Saturating the culture with CO_2_ resulted in a 90% decrease of glycerol, which could be explained in part by the low affinity of form II RuBisCO for CO_2_ (K_C02_ = 0.26 mM). In a second paper, the authors carried out several targeted metabolic engineering to optimize the fermentation kinetic, improving ethanol yield and reducing glycerol production. To achieve this goal, nine copies of the *cbbM* overexpression cassette along with a single expression cassette of *E. coli groEL/groES* chaperones were integrated at the *SGA1* locus using CRISPR-Cas9 tool followed by the integration at X-2 locus of the *PRK1* under the inducible anaerobic *DAN1* promoter. The resulting engineered strain expressing a more robust Calvin-Benson cycle showed significant (31%) reduction of glycerol under glucose anaerobic condition. However, the contribution of the PRK/RuBisCO pathway to NADH reoxidation decreased with increased growth rate, with a concurrent higher contribution of the glycerol pathway. Therefore, a complementary strategy was to abrogate this pathway by removal of glycerol-3-P dehydrogenase encoded by *GPD1* and *GPD2*. While *GPD1* is essential for growth of *Saccharomyces cerevisiae* under osmotic stress (Albertyn et al., 1994), loss of *GPD2* strongly affects growth under anaerobic condition (Ansell et al., 1997). Accordingly, the deletion of *GPD2* in the engineered strain bearing the Calvin cycle resulted in a 62 % decrease of glycerol and 13% increase of ethanol yield as compared to a reference strain. An additional engineering strategy was dedicated to overexpress genes encoding the transketolase and transaldolase with the aim to increase the availability of Ru5P. Indeed, this intermediate is not solely the substrate of PRK, but it is also indirectly implicated in the biosynthesis of nucleic acid, aromatic amino acids and vitamins. Thus, its depletion through PRK could be in part responsible for the poor growth of the strain expressing the Calvin cycle. In accordance with this idea, overexpression of non-oxidative PPP genes *RPEI, TKL1, TKL2, TAL1, RKI1* in the engineered strain also deleted for *GPD2* resulted in a specific growth rate that was virtually the same as the reference strain under anaerobic batch condition on glucose. Furthermore, this engineered strain achieved the best fermentation performance with an increased ethanol yield of 15% and a 90% decrease of glycerol production (Papapetridis et al., 2018).

A comparable strategy has been applied by two other research groups which have equipped a xylose-utilizing *Saccharomyces cerevisiae* with the two Calvin-cycle enzymes PRK and RuBisCO to enhance xylose fermentation with *in situ* CO_2_ fixation (Li et al., 2017; Xia et al., 2017). As *S. cerevisiae* cannot naturally ferment xylose, fungal pathway consisting of a xylose reductase (XR) and xylulose dehydrogenase (XDH) or the bacterial xylose isomerase (XI) have been heterologously expressed, together with upregulation of the native pentose phosphate pathway (PPP) to achieve efficient and rapid xylose fermentation (Matsushika et al., 2009; Young et al., 2010; Oreb et al., 2012). While the XR/XDH pathway appears to be more effective for xylose fermentation rate and ethanol productivity than the XI pathway (Karhumaa et al., 2007), it leads to cofactor imbalance with a surplus of NADH that is regenerated at the cost of by-products formation such as glycerol and xylitol. Expression of the PRK-RuBisCO module should exploit this NADH surplus for recycling CO_2_ that is generated during the fermentation. This strategy was developed by Xia et al. (2017) which used a previously evolutionary engineered *S. cerevisiae* SR8 strain that was able to efficiently co-ferment xylose and glucose. This engineered strain harbored the XR/XDH together with an upregulated PPP and was deleted for *PHO13* and *ALD6* as beneficial targets for xylose fermentation (Kim et al., 2013). Similarly to the work of Guadalupe-Medina et al. (2013), two copies of the *cbbM* gene encoding form-II RuBisCO from *R. rubrum* under the strong *TDH3* promoter and *groEL/groES* cassette under *TEF1* promoter were integrated in the yeast genome (notably at *ALD6* and *PHO13* locus, respectively) leading to a stable platform SR8C^+^ strain. Only RuBisCO activity was detected in yeast strain co-expressing *cbbM* and *groEL/ES*. Then, while the overexpression of PRK from *Spinacia oleracea* alone was toxic for growth on xylose but not on glucose, likely because of ATP depletion or consecutive hyperaccumulation of RuBP, expression of the PRK-RuBisCO module rescued complete growth on xylose, which was accompanied by a 24% reduction of xylitol and 10% increase of ethanol yield while glycerol yield was similar to the control strain. Moreover, it was demonstrated that this engineered *S. cerevisiae* carrying the Calvin-cycle enzymes released 7% less CO_2_ than the control strain during anaerobic xylose fermentation. In a more recent publication, Li et al. (2017) engineered a xylose-utilizing *S. cerevisiae* for co-utilization of maltose, xylose and CO_2_. The rationale behind this strategy was to provide metabolite precursors, energy and reduced cofactor useful for xylose reduction by XR from maltose, which otherwise does not repress xylose fermentation. In addition, a mXR variant that preferred NADH over NADPH was co-expressed together with XR and XDH on a high copy plasmid pRS425. At variance to previous works, genes encoding form-I RuBisCO of *Ralstonia eutropha* or form-II of *R. rubrum*, PRK from *Spinacia oleracea* or *Ralstonia eutrophia* together with *E. coli groEL* and *groES* encoding chaperone were cloned in a low copy plasmid YcpLac33 and transformed into the xylose-utilizing yeast. They reported that these engineered strains grew better on a mix maltose-xylose medium than control strain. They exhibited higher ethanol productivity and yield, with the highest increase of ethanol yield (+ 15%) and sugar consumption rate (+ 63%) with the engineered strain expressing heterotrophic form-I RuBisCO. Using ^13^C-labeled CO_2_ and a metabolic flux index MFI _h−C02_ (Gong et al., 2015) for relative quantification of flux ratio between the CO_2_-fixing by-pass pathway and the central carbon metabolic pathway, an incorporation of 8% CO_2_ into Ru5P was estimated at a rate of 390 ± 50 mg CO_2_/L/h, which is a rate that is in the range of CO_2_ fixation by natural autotrophic microbes (Gong et al., 2015). Altogether, these results indicated that the beneficial effect of implementing the CO_2_-fixation pathway on growth and fermentation largely overcome the additional cost of ATP that is required when this pathway is expressed.

The RuBisCO-based pathway was also investigated in *E. coli* initially as a screen to find out variants of RuBisCO enzyme with higher catalytic activity and better affinity to CO_2_ (Parikh et al., 2006). It was then exploited for *in situ* CO_2_ assimilation in bioproduction (Zhuang and Li, 2013; Gong et al., 2015). For this purpose, the RuBisCO-encoding genes *rbcl-rbcX-rbcS* from the cyanobacterium *Synechococcus sp* PC7002 and PRK-encoding gene from *Synechococcus elongatus* were expressed on a high copy plasmid pET30a and arabinose was provided to the medium as an extra carbon source to enhance C5-phosphorylated sugar required for RuBisCO enzyme. Under this condition, the authors reported that the expression of RuBisCO alone promoted faster and complete consumption of arabinose, whereas the overexpression of PRK alone caused some growth retardation, which was explained by accumulation of RuBP. It was shown that the engineered strain expressing the Calvin-cycle was able to assimilate under anaerobic growth condition CO_2_ at a rate comparable to that of the autotrophic cyanobacteria and microalgae (Gong et al., 2015). However, to overcome the co-cultivation condition, the same authors decided to enhance the non-oxidative pentose phosphate pathway by either overexpressing transketolase (*tktAB*) or by deleting glucose-6-P dehydrogenase (*zwf*) encoding gene. These genetic actions resulted in a significant decrease of CO_2_ yield per glucose consumed. Genes encoding D-lactate dehydrogenase (*ldh*) and fumarate reductase (*frd*) were further deteled in this *zwf* mutant in order to favor recycling of *in situ* CO_2_ into fermentation products ethanol or acetate. However, while this reduced CO_2_ production proved the Calvin cycle pathway to work *in vivo*, and the expression of this cycle did not impede the growth on glucose, only 70% of carbon initially fed in the medium was recovered at the end of the fermentation. Moreover, there was no significant increase in C2-related compounds (Li et al., 2015; Yang et al., 2016). It turned out that this fermentation profile was explained by a huge accumulation of pyruvate that likely resulted from an excessive glycolytic flux at the sugar uptake and phosphorylation step leading to accumulation of NADH and concurrent reduction of pyruvate-formate lyase activity. This activity was inhibited by two concurrent actions, namely a direct inhibition by NADH and a downregulation of *pflB* encoding this enzyme due to the repression of its transcriptional activator encoded by *arcA* (Yang et al., 2016). Intriguingly, these transcriptional effects were already observed upon the simple overexpression of RuBisCO, suggesting a direct impact of the protein on the central carbon metabolism. To overcome these inhibitory problems, pyruvate decarboxylase and alcohol dehydrogenase encoding genes from *Zymomonas mobilis* were overexpressed in the Δ*zwf* Δ*ldh* Δ*frbd* mutant strain equipped with the Calvin-cycle enzymes PRK and RuBisCO. This genetic intervention resulted in a yield of C-2 compounds from glucose 20% higher than that of theoretical fermentation value, which was due to a direct *in situ* CO_2_ recycling into these products by the RuBisCO system. In addition, a rate of 53 mg/L/h of CO_2_ consumption was evaluated, which was in the range of rate measured for CO_2_ fixation by microalgae (Gonzalez Lopez et al., 2009; Ho et al., 2012), arguing that mixotrophic fermentation can be a competitive alternative for reducing loss of CO_2_ and converting it into bio-based products.

## Conclusions and outlook

The carbon management during the metabolic conversion of renewable carbon sources such as sugars by heterotrophic organisms is an attractive, yet partial, solution to combat the acute problem of continuous CO_2_ emission associated with human activities. Since carbon dioxide is an intrinsic by-product of carbon metabolism ensuring the irreversibility of metabolic pathways in which it is involved, the rewiring of carbon metabolism aiming to circumvent this carbon loss must take into account this cost. However, in most of the case that were presented here, this cost can be overridden by the benefit of a higher carbon yield achieved by these refractory or synthetic pathways. This higher carbon yield is the second advantage of this carbon management since it can in principle increase the practical performance of a product per carbon consumed, which is a decisive parameter for the economic evaluation of a biotechnology process. Nonetheless, the synthetic pathways aiming at reducing carbon loss or assimilate CO_2_ are still in their infancy. Substantial work is required to evaluate the potential for industrial application of some of them, such as NOG, MCG, rGS or glycoptimus, whereas others such as the MOG, MSer, and HOB will likely remain as elegant intellectual models illustrating the power of Synthetic Biology in its ability to reformat metabolic pathways.

While heterotrophic organisms are not naturally able to assimilate carbon dioxide, an attractive and complementary strategy to those developed elsewhere to capture CO_2_ is to equip these organisms with the two key Calvin cycle enzymes in order to capture CO_2_ produced during sugar fermentation, and meanwhile use it as an electron acceptor for NADH reoxidation. This dual function has been developed in yeast and *E. coli* and was shown to increase C2- products such as ethanol (Papapetridis et al., 2018; Tseng et al., 2018). The high concentration of carbon dioxide that prevails in industrial fermentations and their usual anaerobic to micro-aeration conditions are factors that should support the proper functioning of this capture system since RuBisCO has low affinity to CO_2_ and its carboxylation activity is antagonized by oxygen. Therefore, this engineering strategy could be implemented without major investment at the industrial scale owing to the fact that GMO's legislation is modified.

Another promising strategy that is already attracting much interest is to exploit the high catalytic capacity of carboxylating enzymes such as the PEP carboxylase to capture “CO_2_” *in vivo*. In addition of engineering specific pathways allowing this capture such as rGS or MCG, a great advantage of using heterotrophic organisms such as yeast or *E. coli* for CO_2_ fixation is their fast growth rates and capability to reach high cell density in bioreactors, both criteria contributing to CO_2_ assimilation rate that are higher than that of auxotrophic organisms. Therefore, engineering heterotrophic microbes for CO_2_ fixation can be a promising avenue to both reduce CO_2_ emission and in the meantime increase carbon yield as the fixed CO_2_ can be easily integrated into the central carbon metabolism. To be even more efficient, the next step will be to employ other energy source than sugar for CO_2_ fixation.

## Author Contributions

All authors listed have made a substantial, direct and intellectual contribution to the work, and approved it for publication.

### Conflict of Interest

The authors declare that the research was conducted in the absence of any commercial or financial relationships that could be construed as a potential conflict of interest.
